# Unbalanced SSFP for super-resolution in MRI

**DOI:** 10.1002/mrm.28593

**Published:** 2020-11-17

**Authors:** Peter J. Lally, Paul M. Matthews, Neal K. Bangerter

**Affiliations:** 1 Department of Brain Sciences, Imperial College London, London, United Kingdom; 2 UK Dementia Research Institute Centre at Imperial College London, London, United Kingdom; 3 Department of Bioengineering, Imperial College London, London, United Kingdom

**Keywords:** spatial encoding, SSFP, structured illumination microscopy, super-resolution

## Abstract

**Purpose:**

To achieve rapid, low specific absorption rate (SAR) super-resolution imaging by exploiting the characteristic magnetization off-resonance profile in SSFP.

**Theory and Methods:**

In the presented technique, low flip angle unbalanced SSFP imaging is used to acquire a series of images at a low nominal resolution that are then combined in a super-resolution strategy analogous to non-linear structured illumination microscopy. This is demonstrated in principle via Bloch simulations and synthetic phantoms, and the performance is quantified in terms of point-spread function (PSF) and SNR for gray and white matter from field strengths of 0.35T to 9.4T. A k-space reconstruction approach is proposed to account for B_0_ effects. This was applied to reconstruct super-resolution images from a test object at 9.4T.

**Results:**

Artifact-free super-resolution images were produced after incorporating sufficient preparation time for the magnetization to approach the steady state. High-resolution images of a test object were obtained at 9.4T, in the presence of considerable B_0_ inhomogeneity. For gray matter, the highest achievable resolution ranges from 3% of the acquired voxel dimension at 0.35T, to 9% at 9.4T. For white matter, this corresponds to 3% and 10%, respectively. Compared to an equivalent segmented gradient echo acquisition at the optimal flip angle, with a fixed TR of 8 ms, gray matter has up to 34% of the SNR at 9.4T while using a ×10 smaller flip angle. For white matter, this corresponds to 29% with a ×11 smaller flip angle.

**Conclusion:**

This approach achieves high degrees of super-resolution enhancement with minimal RF power requirements.

## 1 | INTRODUCTION

The drive toward performing MRI at higher field strengths is largely motivated by the near linear increase in inherent SNR. However, the ability to rapidly generate high SNR clinical images is limited by the specific absorption rate (SAR), which increases at a greater rate. To fully realize the SNR benefits of scanning high field strengths (≥3T), acquisition strategies that overcome traditional SAR limitations while maintaining SNR efficiency are needed.

Steady-state imaging techniques such as balanced steady-state free precession (bSSFP) provide high SNR efficiency, but the associated short TRs used to adhere to acceptable SAR thresholds severely limit the available power for radiofrequency pulses.^1^ If the TR is increased to compensate for high SAR, banding artefacts will become more prominent as the characteristic bSSFP off-resonance profile bands move closer together in frequency. These can obscure important features of interest. As a result, bSSFP sequences have rarely proved to be a practical option for diagnostic imaging at high field, unless 2 or more separate images can be acquired and combined to suppress banding artefacts.^2^

However, rather than treating this characteristic off-resonance profile of bSSFP as a nuisance, many investigators have exploited this to gain additional information (eg, to enhance functional MRI contrast,^3^ to conduct relaxometry or magnetization transfer experiments,^4,5^ or to introduce spectral selectivity into imaging experiments.^6^) There has been limited work exploiting the off-resonance profile of bSSFP to encode additional spatial information into an acquisition, with the exception of the super-field-of-view approach proposed by Lustig et al,^7^ in which the off-resonance profile is used in place of coil sensitivity profiles with a reconstruction similar to that used in parallel imaging.

One way of extending the possibilities of spatial encoding is with the use of “super-resolution” approaches, of the kind originally developed for structured illumination microscopy (SIM)^8,9^ first implemented for MRI by Ropele et al^10^ and recently extended by Hennel et al.^11,12^ In this acquisition scheme, the signal within each imaging voxel is modulated by a characteristic spatial profile. This does not necessarily generate a visible pattern on the acquired image, but allows several images to be acquired separately and merged to achieve a spatial resolution that is greater than the nominal resolution.

Our work proposes using the bSSFP off-resonance profile to conduct super-resolution experiments analogous to those that have been conducted with non-linear SIM. With the combination of short TRs and very low flip angles, an unbalanced SSFP sequence can be used to generate sharp characteristic signal modulations within each imaging voxel. Repeating this process with shifted signal modulation patterns yields a series of nominally lower-resolution images that then can be combined to achieve substantial increases in spatial resolution while maintaining low SAR. We describe this acquisition strategy as comb interleaved excitation and reconstruction (COMBINE). The lower nominal resolution of each acquisition can be exploited to reduce TR, partially offsetting the inherent reduction in SNR efficiency arising from the signal modulation across each voxel. Here, we first outline the approach and describe its sensitivity to typical experimental imperfections, as well as its performance for gray and white matter in field strengths ranging from 0.35T–9.4T. Then, we demonstrate proof-of-concept with both numeric simulations and image acquisition on a physical test object at 9.4T.

## 2 | THEORY

**FIGURE 1:**
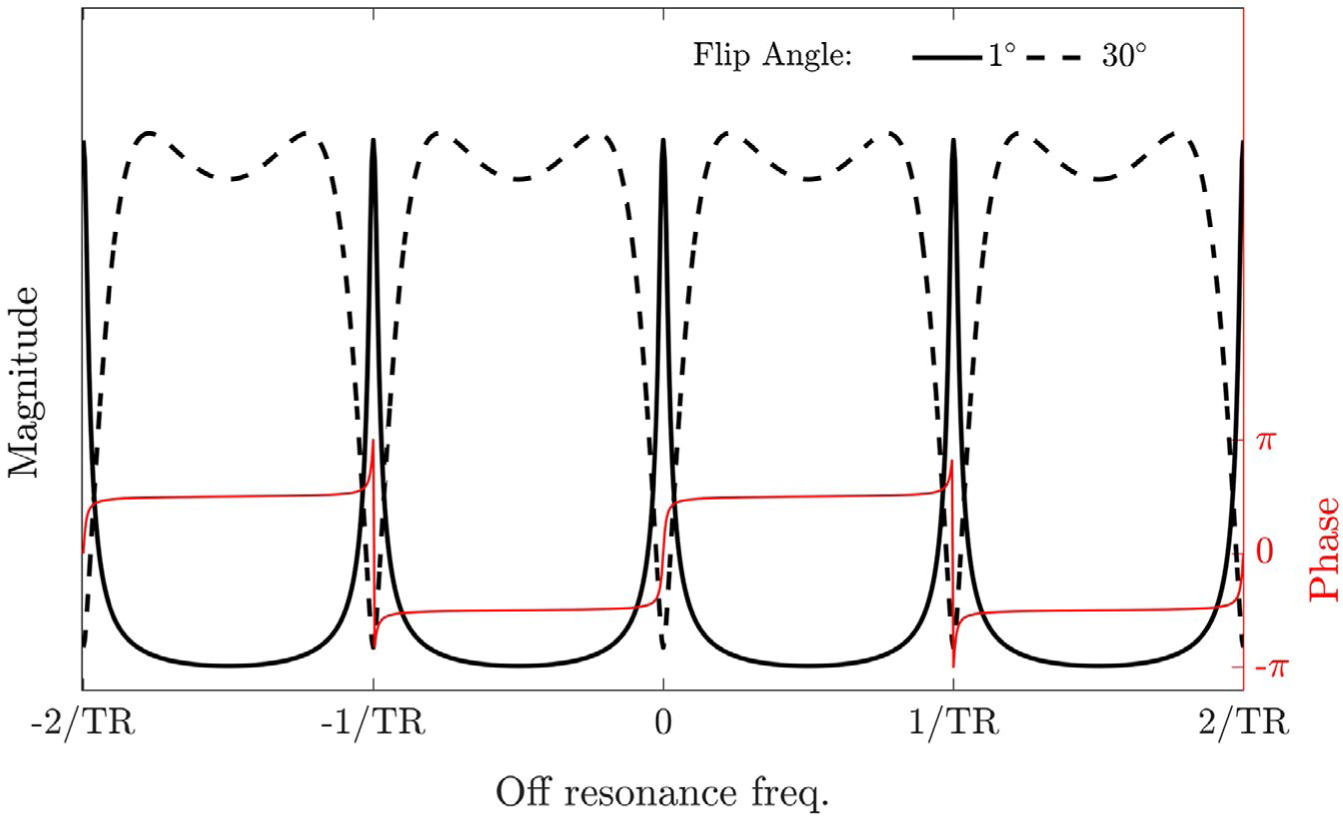
Magnitude of the Bloch-simulated off-resonance profile for a tissue with T_1_ = 600 ms and T_2_ = 100 ms, in a bSSFP acquisition at flip angles of 1° (solid black line) and 30° (dashed black line), with TR = 5 ms. The corresponding phase profile is overlaid in red

The off-resonance profile in bSSFP imaging in a homogeneous medium is well described^13^ and has a periodicity of 2/TR. The magnitude of this approximates a damped comb function in the limit of small flip angles (α < 3°), with peaks at the points of rapid phase transition every 1/TR (Figure 1).

In the presence of an unbalanced linear magnetic field gradient, there is a periodic modulation of the magnetization along 1 dimension of the object, with the magnitude of the steady-state signal concentrated in peaks at the points of rapid phase transition. These points correspond to the regions where banding artefacts are observed on higher flip angle bSSFP acquisitions.

**FIGURE 2:**
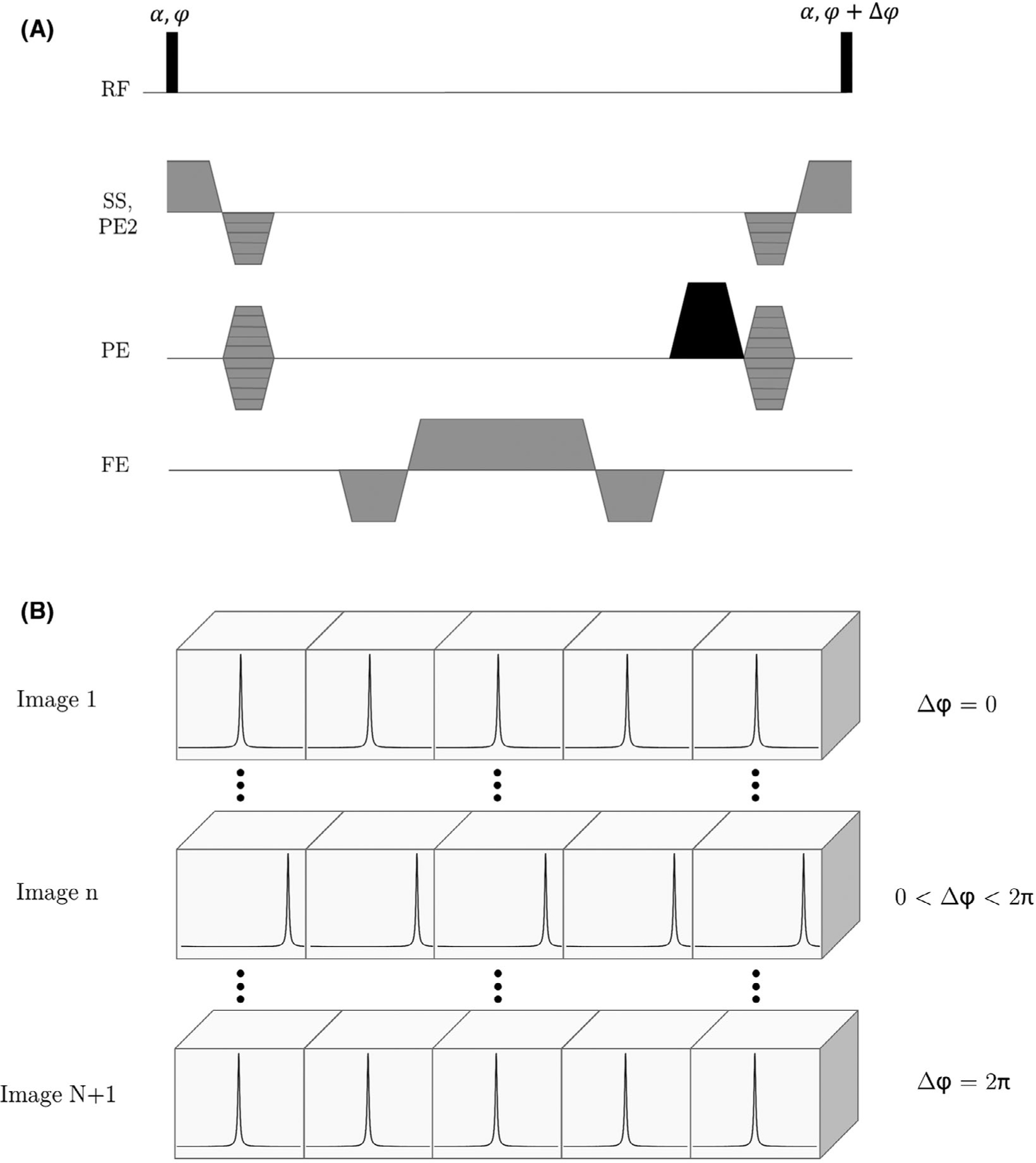
(A) Sequence diagram for the proposed technique, which uses an additional unbalanced spoiler gradient in 1 direction (shaded black). Here, the spoiler gradient is used in the phase encoding direction, but it can be applied along any axis. (B) Schematic of the intravoxel signal modulation during the experiment. Over a series of *N* images, the modulation pattern is swept across the voxel in regular increments. These can then be stitched together to create a super-resolution image

This periodic modulation can be made to coincide with the voxel spacing by ensuring that the unbalanced gradient has an area of 4π*k*_max_∕γ per excitation (eg, in the phase encode direction, Figure 2A), where *k*_max_ is the maximum spatial frequency encoded in the traditional Fourier way, and γ is the gyromagnetic ratio. As a result, the signal from each voxel is predominantly comprised of spins in the locality of the peak in the off-resonance profile (ie, at the center of the voxel).

By applying a phase increment, △*φ*, between successive RF excitation pulses, this intravoxel profile can be offset along the unbalanced gradient direction. Over a series of *N* acquisitions, each with an increasing RF excitation phase increment, a set of low-resolution images is acquired, each of which includes unique high-resolution information about the intravoxel magnetization along a given direction (Figure 2B). This COMBINE imaging experiment therefore follows that of microSPAMM,^10^ but with 3 important differences: (1) the spatial modulation pattern has higher harmonic components; (2) the pattern is generated at the same time as phase encoding; and (3) the radiofrequency pulses have low power.

A super-resolution image can be obtained simply by interleaving the voxels from each low-resolution acquisition and performing a 1-dimensional deconvolution operation. Alternatively, the data can be combined algebraically in the frequency domain.

### 2.1 | k-Space formulation

**FIGURE 3:**
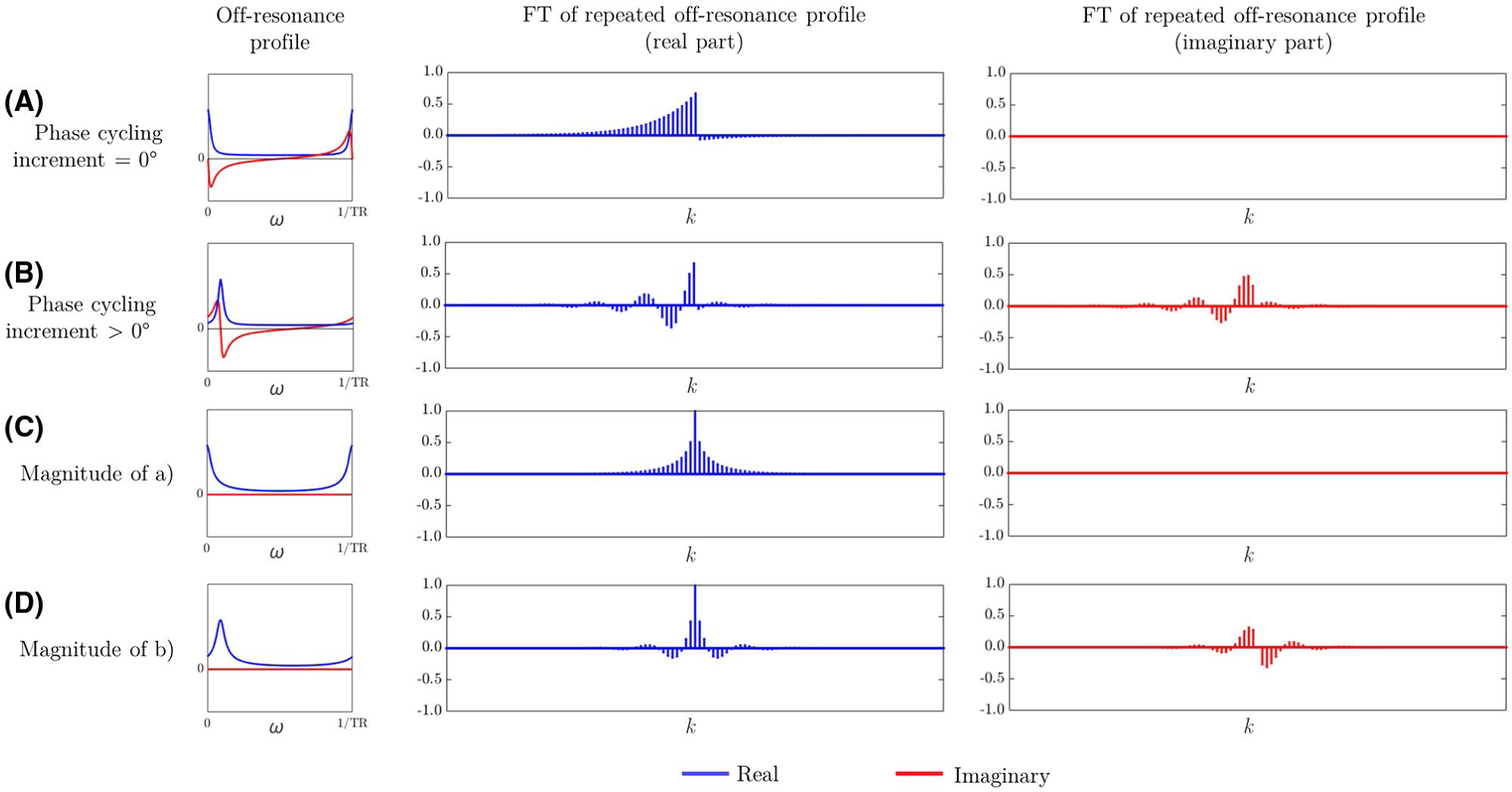
Off-resonance profiles of gray matter at 3T with a 1° flip angle, and corresponding Fourier transforms when this is repeated along a spatial dimension: (A) with the RF phase cycling increment equal to zero; (B) with non-zero linear phase cycling increment; (C) using the magnitude of the off-resonance profile of (A); (D) using the magnitude of the off-resonance profile of (B). Note the marked asymmetry in the k-space harmonics in (A) and (B). Also note the sinusoidal modulation of the harmonics in (B) and (D)

Frequency domain super-resolution COMBINE reconstruction can follow the approach of Hennel et al^11^ but with a damped comb modulation pattern rather than a sinusoid. The Fourier transform of the modulus of the pattern (Figure 3C) is another comb function in k-space, with spacing 2*k*_max_, and with the amplitude decreasing away from the center (Figure 3C). Increasing the sharpness of the spatial comb modulation pattern maximizes the amplitude of these outer harmonic components. Because the pattern is shifted across the voxel, the phase of each of these harmonics in k-space is modulated linearly (Figure 3D). Reconstruction can be performed by solving the system of simultaneous equations for the individual k-space bands and pattern shifts.

By contrast, the Fourier transform of the complex pattern directly results in an asymmetric comb function in k-space (Figure 3A), corresponding to an asymmetry in the configuration states in the extended phase graph formalism.^14^ In this case, the outer harmonic components to one side of the center have markedly higher relative amplitude and so contribute more information to each image. Because the harmonic components on the other side of the center have an amplitude close to 0 in most tissues (apart from where both T_1_ and T_2_ are very long, see Supporting Information Figure S1), they can be omitted from the reconstruction leaving only positive *M*. This asymmetric k-space can be recovered through a similar system of simultaneous equations as described below, and the full super-resolution k-space reconstructed using a partial Fourier approach.

We first describe the acquired “low-resolution” k-space data across *N* pattern shifts as {*s*_1_ (*k*),…, *s*_*N*_ (*k*)}, where *k* describes the dimensions of Fourier spatial encoding. Practically, we construct a matrix Ψ of *M* asymmetric k-space bands and *N* pattern shifts (△*φ*):

(1) $$\Psi =\left[\begin{array}{ccc} e^{-i\Delta \varphi_{1}} & \cdots & e^{-iM\Delta \varphi_{1}}\\ \vdots & \ddots & \vdots \\ e^{-i\Delta \varphi_{N}} & \cdots & e^{-iM\Delta \varphi_{N}} \end{array}\right],$$

and compute the least squares solution for the super-resolution k-space data ***S***, applied to the same coordinate in the low-resolution k-space data across the acquisition series {***s***_**1**_ (*k*), …, ***s***_***N***_ (*k*)}:

(2) $$\Psi \times \left[\begin{array}{c} S\left(k_{x},k_{y}\right)\\ \vdots \\ S\left(k_{x},k_{y}-2Mk_{\max,y}\right) \end{array}\right]=\left[\begin{array}{c} s_{1}\left(k_{x},k_{y}\right)\\ \vdots \\ s_{N}\left(k_{x},k_{y}\right) \end{array}\right],$$

where *k*_*x*_ and *k*_*y*_ describe a single co-ordinate in each low-resolution k-space data set. Here, we have applied the pattern shifts in the *y* direction, and *k*_max,*y*_ describes *k*_max_ along this axis.

The remaining entries of *S* can finally be completed by existing partial Fourier approaches. In the absence of phase instability and B_0_ inhomogeneity, the modulation pattern produces a phase of ~0° in each voxel, and *S* can be simply completed using conjugate symmetry:

(3) $$\left[\begin{array}{c} S\left(-k_{x},-k_{y}+2k_{\max,y}\right)\\ \vdots \\ S\left(-k_{x},-k_{y}+2\text{M}k_{\max,y}\right) \end{array}\right]=\left[\begin{array}{c} S{\left(k_{x},k_{y}-2k_{\max,y}\right)}^{*}\\ \vdots \\ S{\left(k_{x},k_{y}-2\text{M}k_{\max,y}\right)}^{*} \end{array}\right].$$

### 2.2 | B_0_ inhomogeneity

In the presence of B_0_ inhomogeneity the modulation pattern is distorted, with the degree of local distortion in physical space (δ*y*) given by:

(4) $$\delta y=\frac{\gamma }{2\pi }\frac{\partial B_{0}}{\partial y}\Delta y_{nom}TR,$$

where Δ*y*_*nom*_ is the nominal voxel size of the low-resolution images along the chosen super-resolution direction. Therefore, for the same super-resolution voxel size, the degree of distortion increases linearly with increasing low-resolution voxel size, and the gradient of static field inhomogeneities. There is, therefore, a balance between the magnitude of the super-resolution enhancement and the distortion in the final image. Distortion also increases linearly with TR, so this should be reduced as much as possible.

Aside from this distortion, B_0_ inhomogeneity also introduces local phase offsets across the object, which results in local shifts in the off-resonance modulation pattern. This can be corrected with an estimate of the B_0_ field, either derived from a separate acquisition, or from the phase of the data itself.

### 2.3 | Self-navigated B_0_ phase correction

At low flip angles, the peaks of the off-resonance profile occur at points where the phase accumulation is an integer multiple of 2π (Figure 1), and minimal signal contribution arises from magnetization elsewhere in the voxel. Under ideal conditions, the resultant image would, therefore, contain no useful phase information. However, local inhomogeneities in the B_0_ field introduce a phase offset that is proportional to the local shift of the off-resonance pattern. The image phase can, therefore, be used as a B_0_ navigator.

There are several approaches to incorporate this information into the COMBINE reconstruction, and the approach we present here follows that of multifrequency interpolation,^15^ following the suggestion of Hennel et al.^12^ Practically, we introduce different constant phase offsets in the raw k-space data to simulate separate reconstructions at a range of frequencies, and choose the reconstruction that provides the optimal image quality metric on a voxel-wise basis.

**FIGURE 4:**
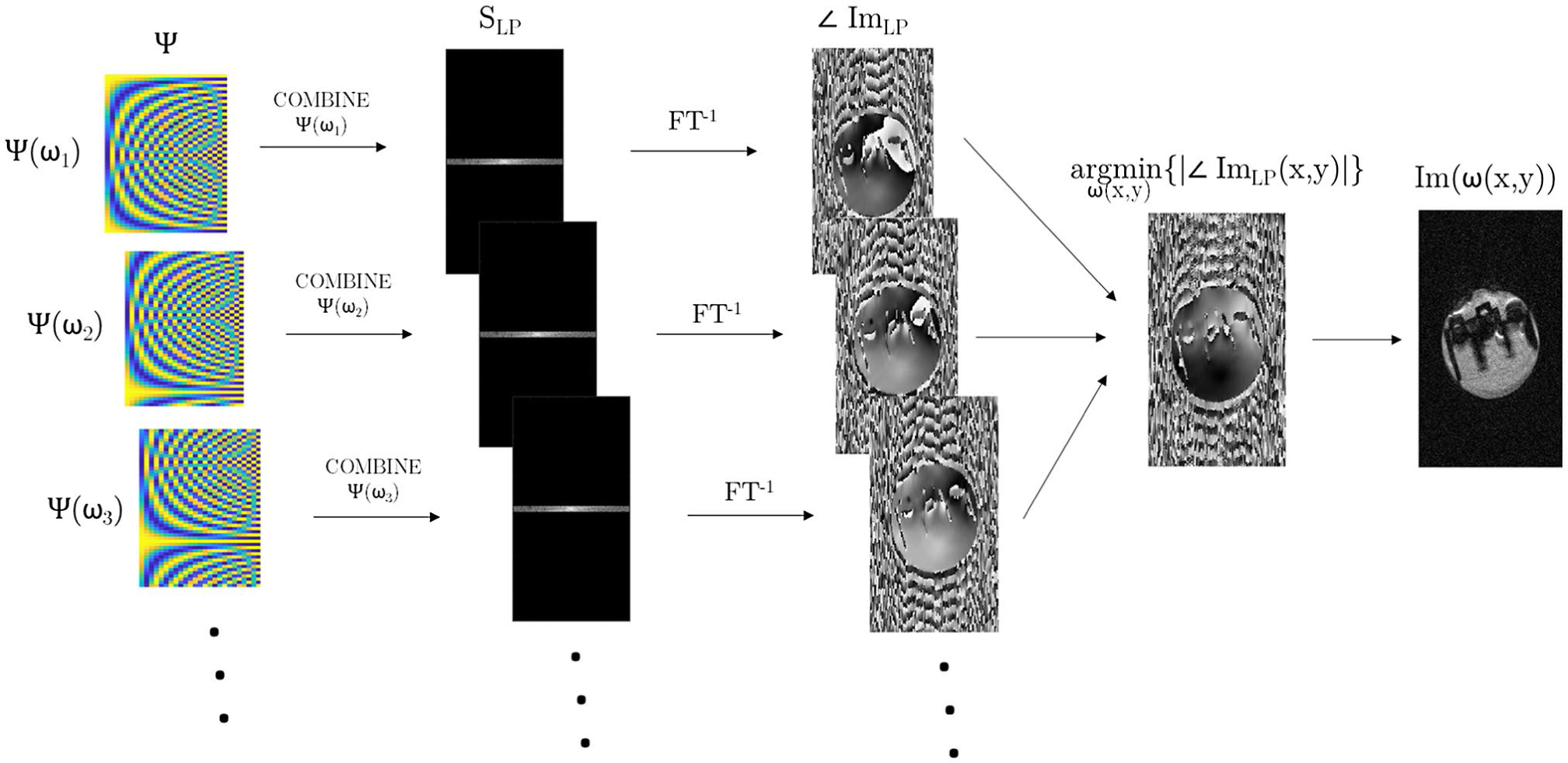
Illustration of the multi-frequency reconstruction procedure. A phase offset it is introduced to Ψ to mimic a local offset frequency ω, and a low-pass image (Im_LP_) is obtained by zero-filling a single band of k-space data (S_LP_) and performing the inverse Fourier transform. The phase of this image is calculated, and the process repeated for a range of offset frequencies. A map of ω is obtained by finding the value that minimizes the absolute phase on a voxel-wise basis. Finally, full-resolution images are reconstructed at each of the same offset frequencies, and the reconstruction at the optimal ω is chosen on a voxel-wise basis

At low flip angles, we can simply choose the reconstruction that minimizes the absolute phase in each voxel, as illustrated in Figure 4. To minimize the effects of noise, and making the assumption that the phase is slowly varying in space, we estimate the phase from the zero-padded central band of k-space.

### 2.4 | $B_{1}^{+}$ inhomogeneity and relaxivity dependence

**FIGURE 5:**
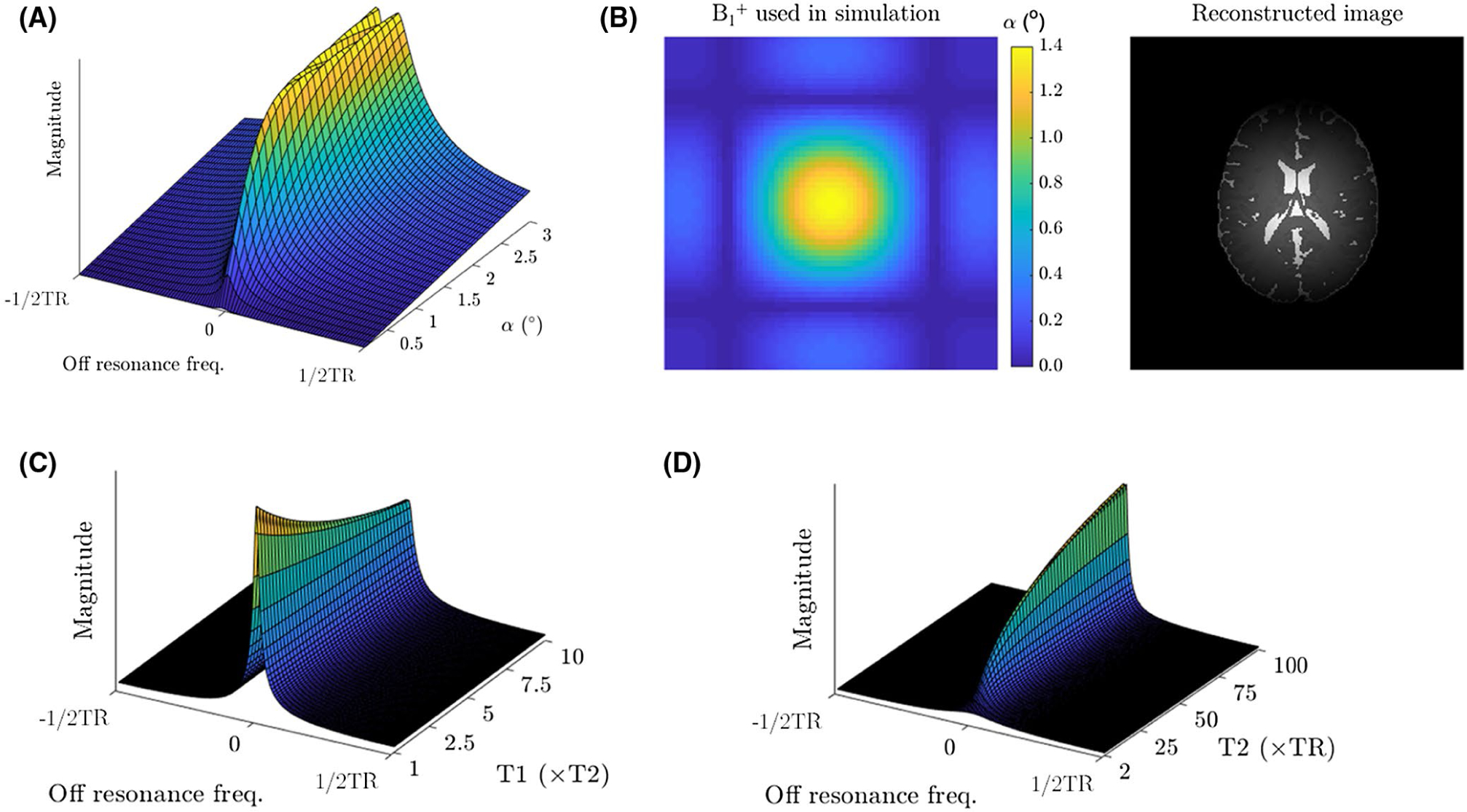
(A) Flip angle dependence of the bSSFP signal profile, for tissue parameters of T_1_= 600 ms and T_2_= 100 ms, and TR = 5 ms. (B) Bloch simulation of COMBINE in a numeric brain phantom, in the presence of spatial ${\text{B}}_{1}^{+}$ in homogeneity. (C) T_1_ dependence of the bSSFP signal profile, for a flip angle of 1°, TR = 5 ms, and T_2_= 100 ms. (D) T_2_dependence of the bSSFP signal profile, for a flip angle of 1°, TR = 5 ms, and T_1_ = 600 ms

Whereas in the very low flip angle regime (α ≤ 1°), ${\text{B}}_{1}^{+}$ inhomogeneity produces an approximate scaling of the magnitude of the off-resonance profile (Figure 5A). This manifests primarily as signal non-uniformity in the resultant image (Figure 5B).

In the regime where α > 1°, the sharp profile spike begins to split and follows an increasingly pronounced bimodal distribution: there is a decrease in signal magnitude at the center frequency with signal maxima immediately surrounding the main peak (Figure 5A). ${\text{B}}_{1}^{+}$ inhomogeneity in this regime produces a spatially variant point spread function in the resultant image. However, if an accurate ${\text{B}}_{1}^{+}$ map has been acquired, this information could be incorporated into a final deconvolution step.

This technique is ideally suited to rapid scanning with α ≤ 1°, because the low SAR enables TR to be as short as the gradient hardware will allow. In this scenario, the relaxivity dependence is then dominated by T_2_ effects and relatively insensitive to T_1_ effects (Figure 5C,D). As a result, the images generated by this approach have a mixture of proton density and T_2_-weighting. This also results in a tissue-dependent point spread function that is predominantly affected by T_2_.

### 2.5 | SNR efficiency

**FIGURE 6:**
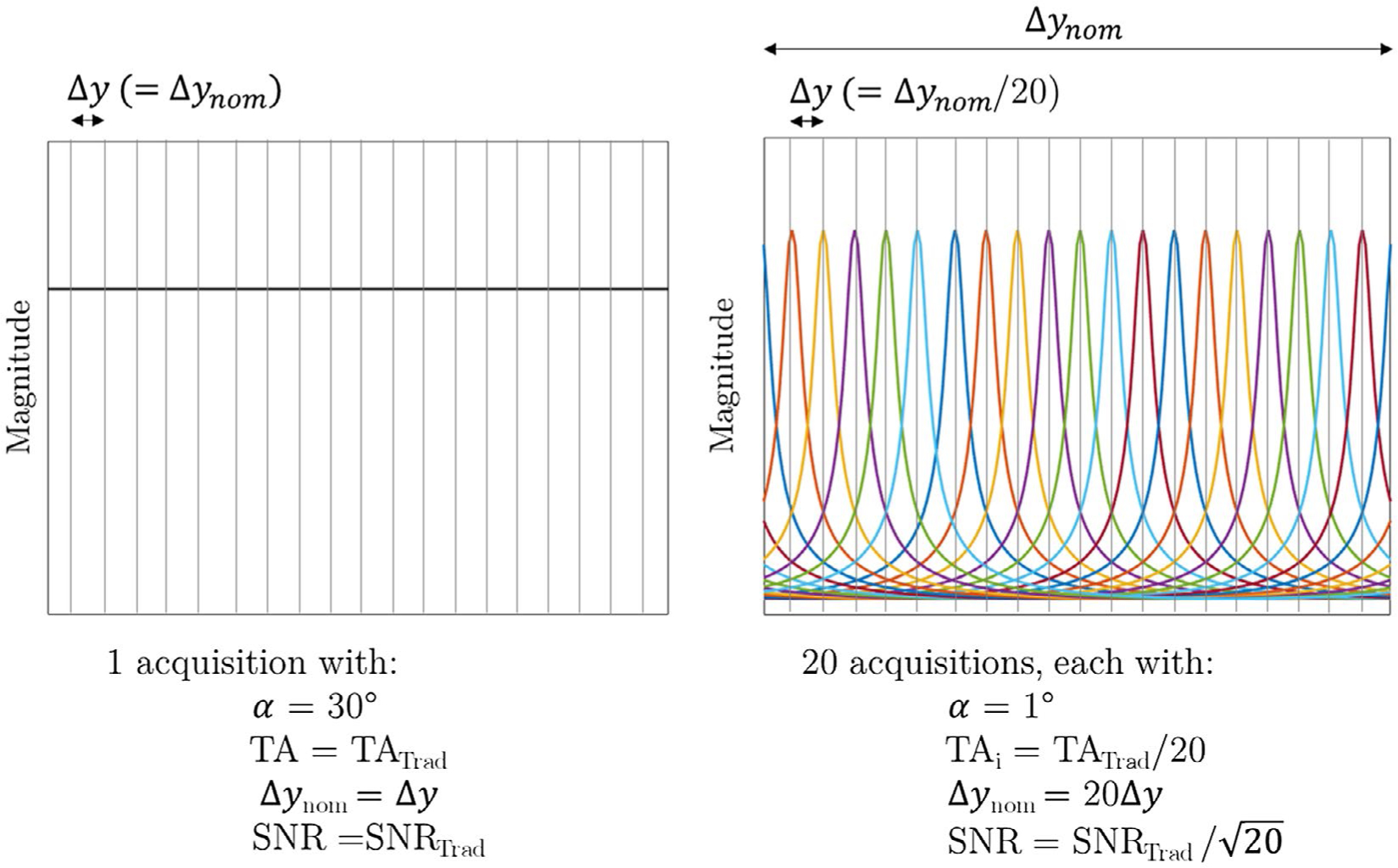
Illustration of the efficiency considerations when comparing the approach to traditional GRE sequence with the same TR of 5 ms. Left: the GRE signal is assumed to be perfectly spoiled so that the magnitude in each voxel (with nominal voxel size Δ_*ynom*_ ≡ Δ_*y*_) is the mean of the bSSFP profile at 30°, and results in a total acquisition time of TA_Trad_. Right: illustration of the proposed approach with a resolution enhancement factor of 20, determined by the FWHM of the off-resonance profile. In this case, the nominal voxel size (Δ*y*_*nom*_) is 20 times larger than the desired super-resolution voxel size (Δ*y*_)_, and each image is acquired 20 times faster. The full image is acquired in the same total time (because TR is equal), and the integral of the magnitude signal in the final image is near equivalency, but there is an SNR efficiency penalty of approximately $\sqrt{20}$

Under appropriate conditions COMBINE can be a reasonably SNR efficient spatial encoding strategy, as the maximum of the off-resonance profile low-flip angle regime is close to that of the pass band at the higher flip angles typically used in bSSFP imaging (Figure 1). By choosing acquisition conditions such that the width of the off-resonance peaks approximates that of the desired high-resolution voxel dimension, the SNR of the final image (SNR_SR_) can be expressed in terms of the SNR of the equivalent spoiled gradient echo image acquired through traditional spatial encoding at a higher flip angle (SNR_GRE_):

(5) $$SNR_{SR}=\frac{SNR_{GRE}}{\sqrt{\Delta y_{nom}/\Delta y}}\frac{\left|\int_{0}^{\Delta x}\Gamma_{SSFP}(y)\partial y\right|}{\left|\Gamma_{GRE}\Delta y\right|},$$

where Δ*y*_*nom*_ is the nominal voxel size of the low-resolution image, Δ*y* is the desired high-resolution voxel size, and Γ is the spatial modulation of the signal across *y* for each sequence (and is constant for gradient echo [GRE]). For the proposed low flip angle unbalanced SSFP technique, the integral of the off-resonance profile across Δ*y* is approximately equal to that from the GRE signal at higher flip angles (ie, the mean of the off-resonance bSSFP profile, Figure 6), and so Equation (5) reduces to:

(6) $$SNR_{SR}\approx \frac{SNR_{GRE}}{\sqrt{\Delta y_{nom}/\Delta y}}.$$

In comparison with traditional phase encoding, SNR efficiency is, therefore, reduced by approximately the square root of the enhancement factor because of the acquisition of fewer phase encode lines per low-resolution image (Figure 6). In the case in which the low-resolution image comprises a single line, the SNR efficiency is equivalent to that achieved when Δ*y*_*nom*_∕Δ*y* slices are scanned sequentially instead of a 3D scan with the same TR. However, the required RF power is dramatically reduced.

Equation (5) and Figure 6 provide an intuitive understanding of the approach where voxels are simply interleaved to achieve super-resolution. However, this discards much of the spatial information encoded in the off-resonance profile. The SNR and point-spread function (PSF) can be better optimized by considering the off-resonance profile of specific tissues of interest, and choosing *M* (the number of asymmetric k-space bands) and *N* (the number of independent low-resolution images) accordingly in the k-space formulation (Equation 2).

### 2.6 | Optimal choice of *M* and *N*

**FIGURE 7:**
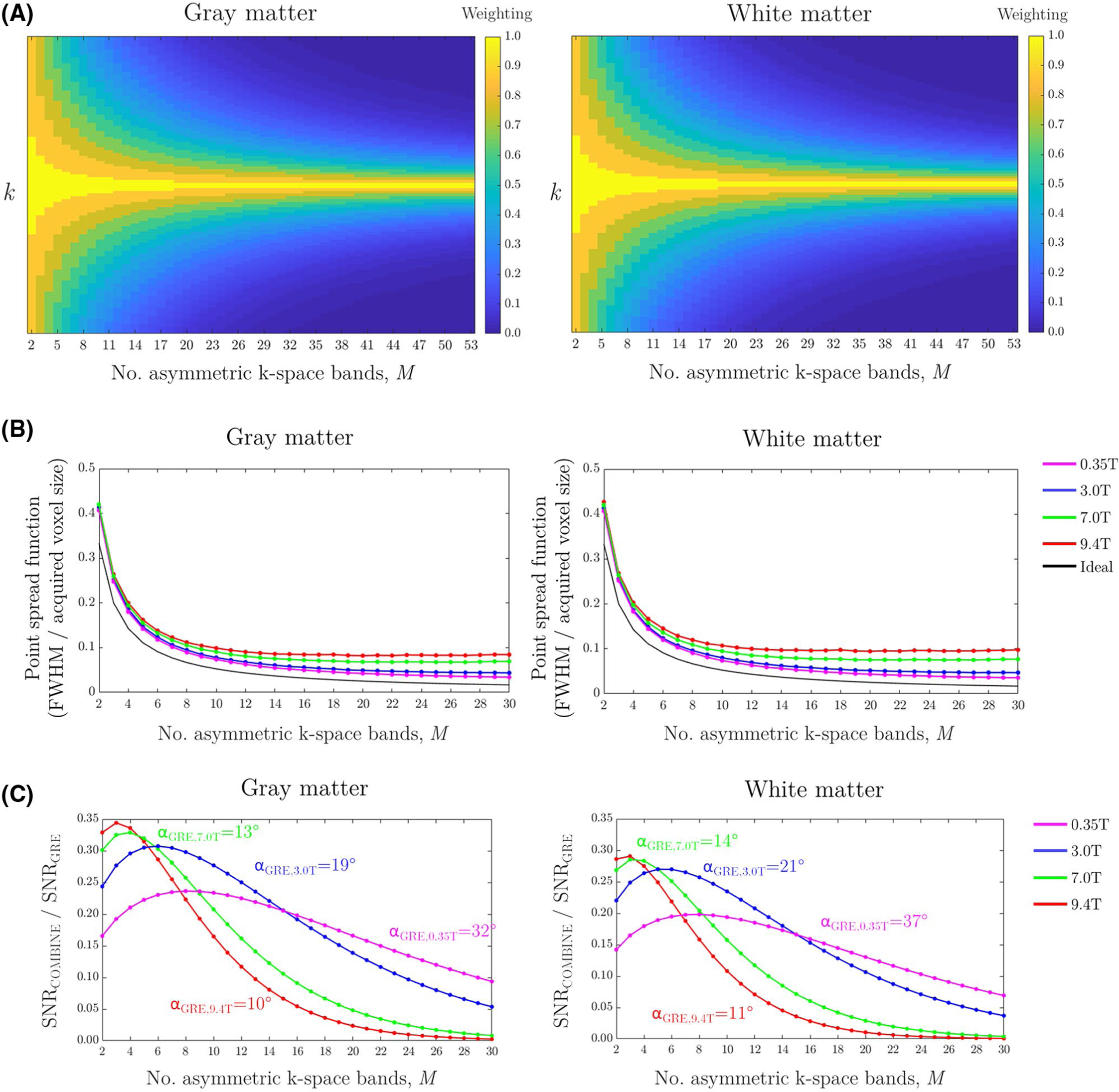
(A) Bloch simulated k-space weighting for gray matter (left) and white matter (right) with a flip angle of 1°, using T_1_ and T_2_ from Zhu et al,^16^ and with increasing *M*. (B) Bloch simulated point-spread function for gray matter (left) and white matter (right) at different field strengths with a flip angle of 1°, using T_1_ and T_2_ from Zhu et al,^16^ and in comparison to the ideal voxel size. (C) SNR of COMBINE with a flip angle of 1° in comparison to a partial-Fourier gradient spoiled*M-*segment acquisition at the optimal flip angle and TE = 0, for T_1_ and T_2_of gray and white matter observed at different field strengths.^16^ Both acquisitions use the same arbitrary TR of 8 ms, and record the same number of k-space lines. This excludes any aliasing effects during the COMBINE reconstruction

Equation (2) implicitly assumes that the harmonics in the off-resonance profile have equal amplitude and extend infinitely in k-space. However, as shown in Figure 3, the amplitude of each harmonic reduces away from the center of k-space because of the damping profile of the excited comb, which is largely influenced by tissue T_2_, and introduces a relative weighting of the signal in different k-space bands (Figure 7A). This creates a degree of low-pass filtering in the reconstructed images, and determines a limiting width of the PSF with increasing *M*. The impact of *M* on PSF is shown for both gray and white matter in Figure 7B at different magnetic field strengths, using T_1_ and T_2_ estimates from Zhu et al.^16^

The implicit low-pass filtering of the COMBINE data in k-space leads to difficulties in directly comparing its SNR performance with traditional spatial encoding. Instead, we can scale the data in each k-space band to reverse the low-pass filtering of the signal so that the differences in SNR performance can be assessed by simply comparing the propagation of noise, as proposed in Hennel et al.^11^ To do this, we modify Equation (2) by introducing the *M* × *M* matrix Σ_**comb**_ that contains only the harmonic amplitudes along its diagonal, and produces the observed low-pass filtering.


(7) $$\Psi \sum_{\text{comb}}S=S.$$

The least squares solution is then given by:

(8) $$S=\sum_{\text{comb}}^{-1}{\left(\Psi^{*}\Psi \right)}^{-1}\Psi^{*}s={\text{N}}^{-1}\sum_{\text{comb}}^{-1}\Psi^{*}s.$$

Because the noise in each measurement is equal and uncorrelated (described by $\sigma_{s}^{2}$), the expression for the reconstructed image variance after Fourier transformation is given by:

(9) $$\begin{array}{l} \sigma_{\text{comb}\;}^{2}=\mathrm{tr}\left(\bar{SS^{*}}\right)=\mathrm{tr}\left(\left({\text{N}}^{-1}\Sigma_{\text{comb}\;}^{-1}\Psi^{*}\right)\left({\text{N}}^{-1}\Psi \Sigma_{\text{comb}\;}^{-1}\right)\right)\sigma_{s}^{2}\\ \;={\text{N}}^{-1}\mathrm{tr}\left(\Sigma_{\text{comb}}^{-2}\right)\sigma_{s}^{2}. \end{array}$$

The noise is, therefore, colored, as it is weighted in each k-space band by the inverse of the corresponding harmonic intensity. In comparison, a traditional gradient spoiled acquisition comprising *M* segments with the same TR, same asymmetric k-space coverage and readout as used in COMBINE has image variance of:

(10) $$\sigma_{\text{trad}\;}^{2}=M\sum_{0,\mathrm{trad}}^{-2}\sigma_{s}^{2},$$

where the scalar Σ_0,trad_ describes the spatially uniform signal scaling in the gradient spoiled image (for a given tissue), given by the amplitude of its zeroth harmonic. This is equivalent to normalizing Σ_**comb**_ to produce an image with the same signal scaling as the traditional gradient echo acquisition.

In COMBINE, the number of acquired images *N* must be greater than or equal to *M*. The most SNR efficient experiment would use *N* = *M*, but if there is redundancy in the acquisition the SNR scales as *N*^1/2^, as with traditional image averaging. Although more SNR efficient, a small *N* will introduce aliasing from higher order harmonics during the COMBINE reconstruction, producing additional variance in the final images that is not accounted for in Equation (9).

The effect of choosing different *M* is shown by direct comparison with a gradient spoiled acquisition comprising *M* segments, as shown in Figure 7C for gray and white matter at different field strengths. Here, the TR of both acquisitions is fixed at 8 ms, with the COMBINE flip angle fixed at 1° and the gradient spoiled flip angle set at the optimum for the tissue and field strength (ranging from 10°–37°), and the relative SNR is calculated from the ratio of Equations (9) and (10). Although there is a reduction in SNR with COMBINE, this may be less than one would expect if using flip angles that are an order of magnitude smaller than the optima for gradient spoiled imaging.

## 3 | METHODS

Simulations were performed on a numeric phantom using the parallelized version of JEMRIS (“pjemris”, v2.8.2),^17^ implementing a 2D bSSFP sequence with an additional unbalanced spoiler gradient on the phase encode axis, as shown in Figure 2A. The sequence parameters were as follows: TR/TE = 8/4 ms; N_PE_ × N_FE_ = 17 × 289; FOV = 289 × 289 mm^2^; slice thickness = 1 mm; α = 0.1°. The first low-resolution image was generated by setting the RF excitation phase increment to 0°, and subsequent images were acquired with the phase increment increased in steps of (360/17)°. At the same time, the receiver phase was adjusted to ensure that the modulation pattern remained fixed relative to the imaging grid. This process provided a series of 17 low-resolution images with evenly spaced modulation patterns across the object. To reconstruct the super-resolution images, the magnitude was taken of each low-resolution image, and the voxels interleaved in the image domain. For simplicity, no deconvolution operation was performed.

The numeric brain phantom was rendered with a resolution of 289 × 289 isochromats and with 3 tissue compartments: CSF with T_1_ = 2569 ms, T_2_ = 329 ms, proton density (PD) = 100%; gray matter with T_1_ = 1331 ms, T_2_ = 80 ms, PD = 86%; and white matter with T_1_ = 832 ms, T_2_ = 110 ms, PD = 77%.^18^ During this acquisition, both B_0_ and B_1_ were set to be perfectly homogeneous.

Physical experiments were then performed on a 9.4T Bruker BioSpec 94/20 (Bruker, Karlsruhe, Germany) equipped with a transmit-receive volume coil with an inner diameter of 40 mm. A Lego brick was submerged in water doped with copper sulphate inside a cylindrical sample tube and imaged at isocenter with the vendor’s 2D bSSFP sequence, modified to include an unbalanced spoiler gradient in the phase encode direction. The sequence parameters were as follows: TR/TE = 5/2.5 ms; N_PE_ × N_FE_ = 32 × 128; FOV = 58 × 40 mm^2^; slice thickness = 1.2 mm; α = 0.4°; 1000 dummy TRs (5 s of steady-state preparation); 36 separate images at equidistant phase increments. This process provided a series of 36 low-resolution images (1.8 × 0.3 mm^2^) with evenly spaced modulation patterns across the object. The super-resolution image was reconstructed via the multi-frequency reconstruction previously described, with 100 equidistant phase offsets between 0 and 2π. A separate 3D B_0_ map was acquired using the vendor’s standard sequence, with an isotropic resolution of 0.9 mm. This was used for reference only, and was not incorporated in the reconstruction.

## 4 | RESULTS

**FIGURE 8:**
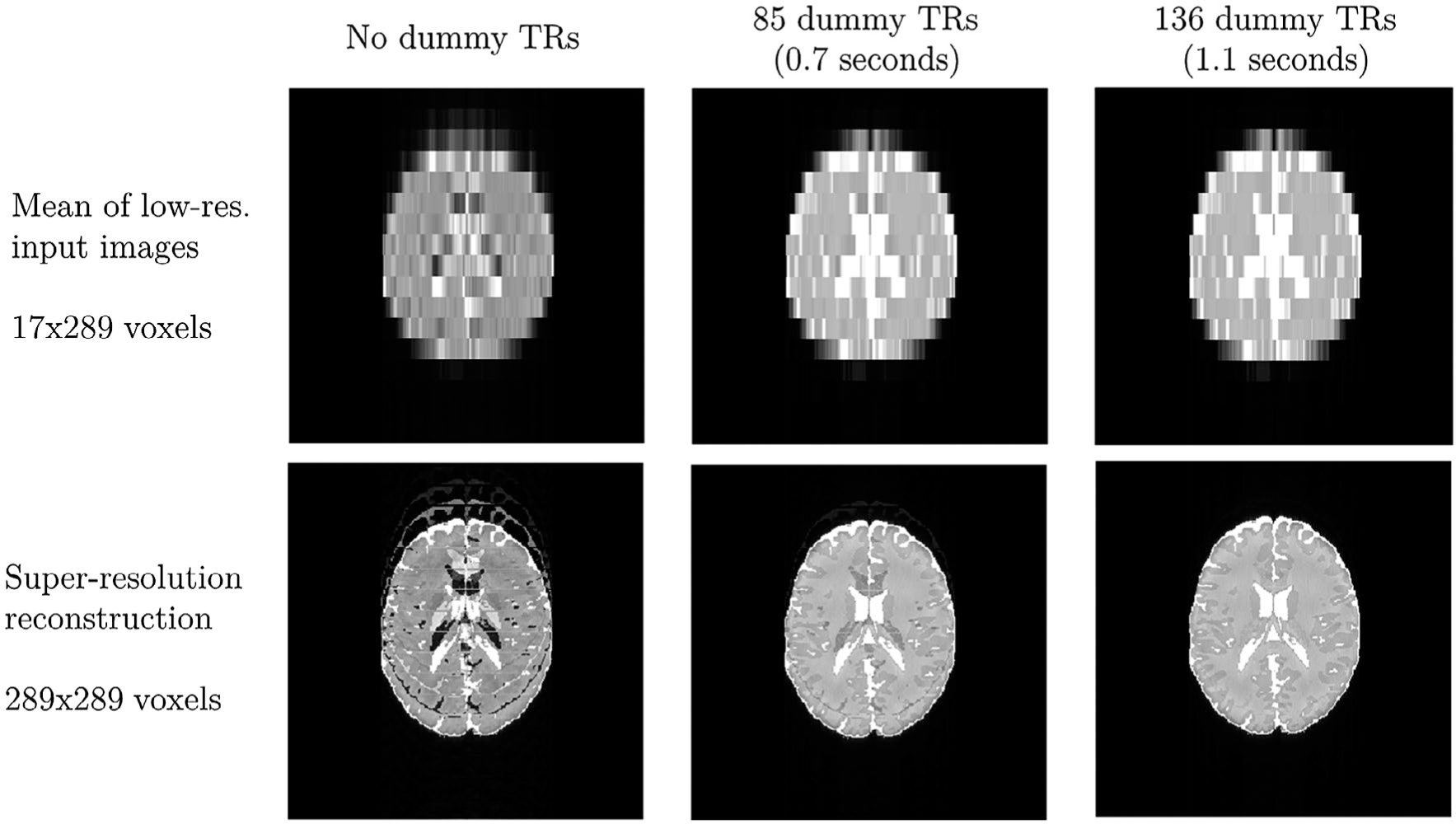
Bloch simulations on a numeric brain phantom, applying the proposed super-resolution approach to generate a 289 × 289 image from 17 low-resolution 17 × 289 images. As the number of dummy TRs are increased from left to right, the reconstruction artefacts decrease

The results of Bloch simulations for COMBINE are shown in Figure 8. Where the magnetization has not yet reached the steady state when acquiring each low-resolution image, the super-resolution reconstruction results in artefacts. However, these artefacts can be largely suppressed by inclusion of a preparation period; with the numeric phantom used, 1.1 s (the equivalent of 8 low-resolution acquisitions) for each of the 17 low-resolution images is adequate for complete suppression. Whereas the transient period of bSSFP is typically affected by rapid oscillations in the absence of catalyzed excitation schemes (eg, preparation with a ramped flip angle^19^), this is absent when such a low flip angle is used (Supporting Information Figure S2).

**FIGURE 9:**
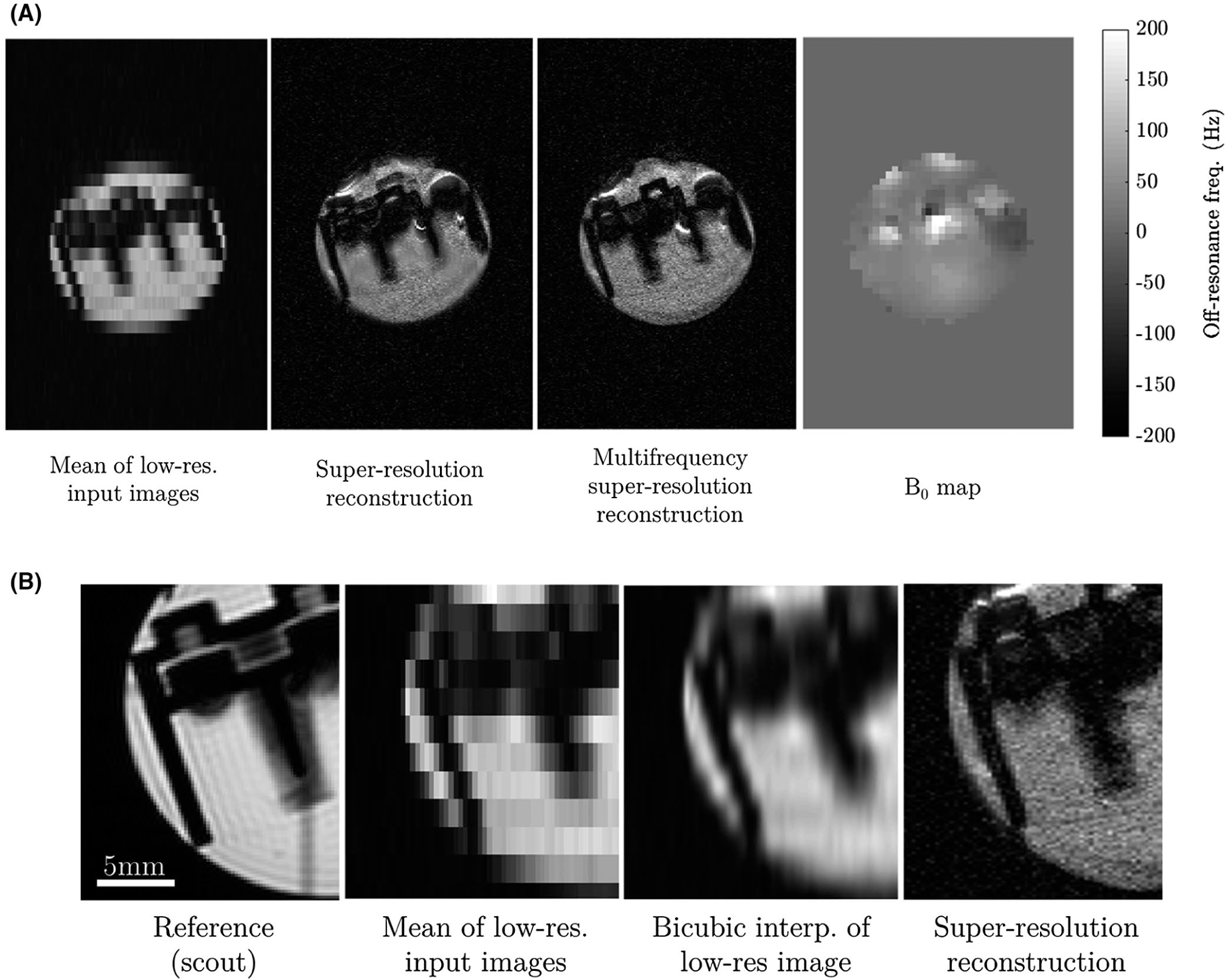
(A) Demonstration of the super-resolution approach in the physical phantom, including (from left–right): the mean of the low-resolution input images; single frequency super-resolution reconstruction; multifrequency super-resolution reconstruction; vendor-provided B_0_ map (that was not used in the reconstruction but is provided for reference) indicating the presence of substantial static field inhomogeneities. (B) Close-up of the images obtained from the physical phantom, including (from left–right): a reference structural scout image; the mean of the low-resolution input images; a bicubic interpolation of the mean of the low-resolution input images; and the proposed multifrequency super-resolution reconstruction. The proposed approach clearly enhances spatial resolution in comparison to straightforward image interpolation

The physical phantom experiment provided proof-of-principle for the super-resolution reconstruction approach, with a clear improvement in the ability to visualize the phantom structure. Despite the multi-shot nature of the acquisition, the resultant image also shows homogeneous signal intensity across the object. Although there were clear distortion effects because of B_0_ inhomogeneities (>200 Hz, Figure 9A), a multi-frequency reconstruction effectively suppressed these.

The simple super-resolution approach provided clear improvements in spatial resolution over the low-resolution input images, as well as when compared to a straightforward bicubic interpolation (Figure 9B).

**FIGURE 10:**
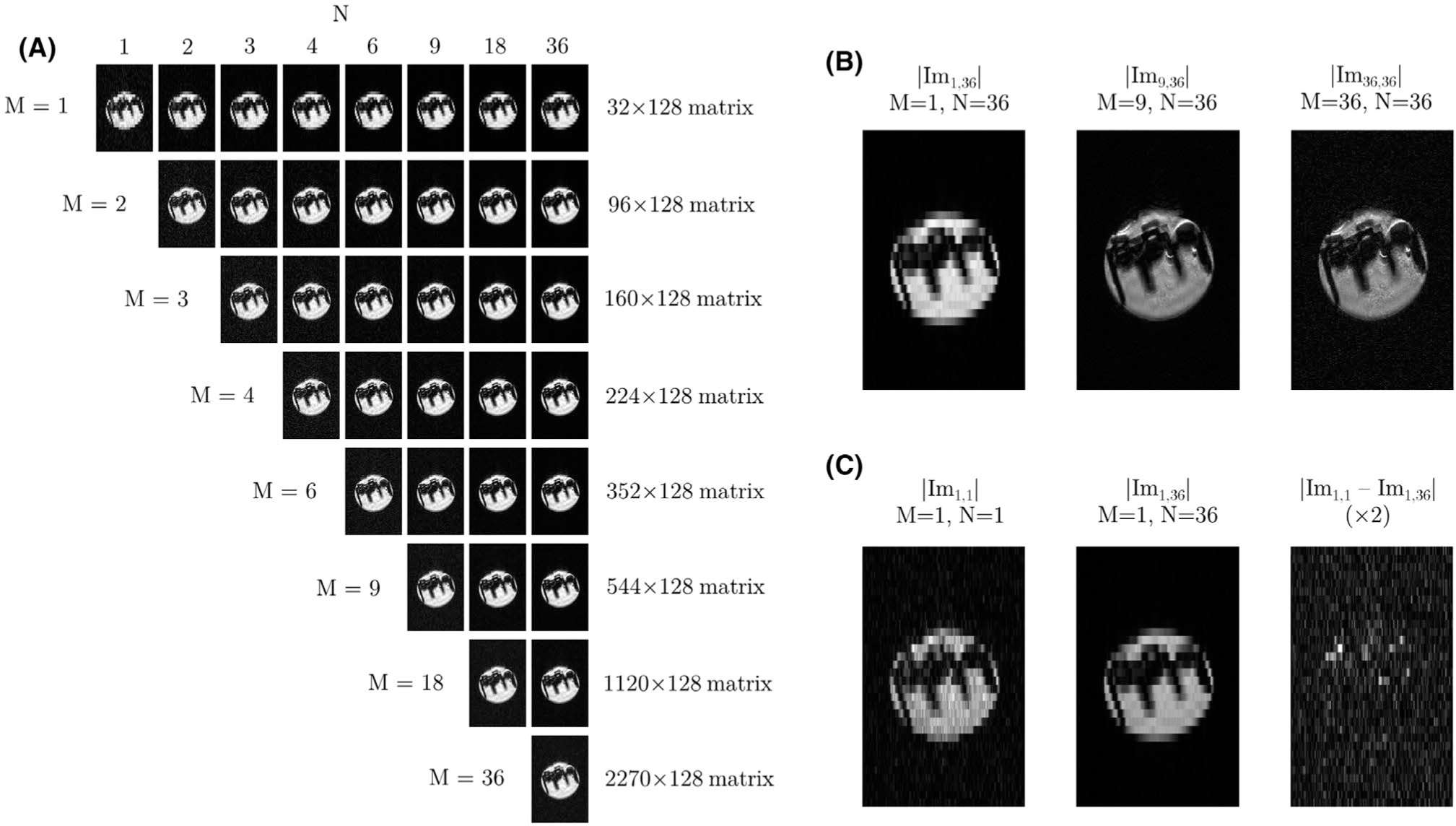
(A) Reconstructions at a single frequency from different combinations of *M* and *N* from a COMBINE acquisition of 36 images at equidistant phase cycling offsets. Each reconstruction produces a matrix size that is 2*M*-1 times larger along the super-resolution dimension. (B) For the same number of input images (*N*= 36), increasing *M* improves the spatial resolution of the resultant image and reduces the SNR. For large *M*, the additional reconstructed k-space bands contribute more noise than useful high-resolution information. (C) The difference of 2 acquisitions with the same *M* but different *N* (*N*= *M*= 1; *M*= 1, *N*= 36). The subtraction image shows that aliasing produces structured variance, localized to regions where there is a significant local B_0_ offset because of eg, air bubbles. Outside of these regions the aliasing effects are benign

The effect of reconstructing an increasing number of asymmetric k-space bands, *M*, from the same data set is shown in Figure 10A,B, for a single offset of the multi-frequency reconstruction. For increasing *M* the spatial resolution increases and SNR reduces, but for large *M* the outermost k-space bands contribute more noise than useful signal. In Figure 10C, the image from *M* = *N* = 1 is subtracted by the equivalent image with *M* = 1, *N* = 36. In the former image, *N* is fewer than the number of harmonic components and results in aliasing during the reconstruction, whereas the aliasing is suppressed in the latter image. The subtraction image is largely noise-like, but there are subtle artifacts localized to regions with a significant local B_0_ offset. This necessitates the use of the proposed multi-frequency reconstruction.

## 5 | DISCUSSION AND CONCLUSIONS

This work demonstrates the feasibility of using the off-resonance magnetization profile in steady-state imaging to achieve super-resolution in MRI. The COMBINE approach requires very low radiofrequency power and so overcomes the typical limitations on SAR imposed at high field (≥3T), which could be leveraged to reduce the TR and offset the inherent loss of SNR efficiency.

If using COMBINE with *M* k-space bands the maximum combined phase encoding rewinder and spoiler gradient area is reduced by a factor of (2*M*-1)/3 compared to a traditional phase encoding rewinder. The corresponding factor for the preceding phase encoding gradient is (2*M*-1). For *M* = 3, this reduces the total phase encoding gradient area by a factor of 2.5 in the super-resolution direction.

The proposed approach shares similarities with other super-resolution techniques,^10–12^ but is specific to the SSFP sequence. Other approaches introduce the signal modulation as a preparation module so that this can be incorporated into various acquisition strategies, whereas in this case the modulation is caused by the inherent off-resonance profile of bSSFP. This means that COMBINE is less flexible in terms of combining it with other readout schemes, but allows the rapid collection of high resolution images. In addition, the modulation pattern contains higher harmonic components that enable a greater degree of resolution improvement for the same nominal resolution. In some respects, COMBINE has similarities to through-plane super-resolution techniques such as gSlider,^20^ because high-resolution data are reconstructed from a linear combination of low-resolution samples with an encoded spatial interference pattern.

Although a ${\text{B}}_{1}^{+}$ map was not collected as part of the physical experiment, it is expected that inhomogeneity of the RF transmit field will have an important impact on images. However, in the current experiment images displayed reasonable homogeneity across the phantom. Because future work extends this super-resolution approach to other contexts, care will need to be taken because of the risk that the prescribed flip angle may not be accurately delivered because of non-linear performance of RF amplifiers in the low flip angle range.^21^ Bespoke ${\text{B}}_{1}^{+}$ mapping strategies will, therefore, need to be used, particularly when α ≥ 1°, otherwise deconvolution approaches for inhomogeneity correction will fail.

The phantom experiments used flip angles in the range of α < 1° to capitalize on the associated low SAR and sharp spectral profiles. Because the profile approximates a damped comb function, it is close to the ideal sampling pattern of the adjacent bands of k-space. Each image is, therefore, a weighted sum of successive bands of k-space, with the weighting factor determined by the intensity of the harmonic components. The technique could be readily extended to higher flip angles, but this comes with 2 important caveats: (1) the integral of the complex off-resonance signal profile across the voxel becomes more complicated, necessitating a complex deconvolution step using a good estimate of the ground truth; and (2) there are fewer high order harmonic components in the off-resonance profile, and these have reduced intensity—this limits the extent of super-resolution enhancement and further magnifies noise away from the center of k-space. Provided these limitations can be mitigated well, applying this technique at higher flip angles could provide SNR efficiency improvements.

Another interesting extension will be to leverage the short TRs of the proposed acquisition scheme to examine tissues with short T_2_s. However, as T_2_ approaches TR the spins exhibit a broader off-resonance profile (Figure 5D) that would necessitate the use of different deconvolution kernels for different tissues. This could surpass the spatial resolution limits imposed by blurring because of rapid ${\text{T}}_{2}^{*}$ decay, but the degree of spatial resolution increase would be ultimately limited by the width of this broader off- resonance profile.

One limitation of the use of SSFP sequences is the required preparation time for the magnetization to build up to the steady state. Here, the use of low flip angles could enable imaging even in the transient state, reducing this preparation time. In the 2D experiments presented here, the preparation time far outweighed the acquisition time for each image and so the acquisition strategy was inefficient, but this will not be the case for 3D imaging where the preparation time will add only a minimal increase to the acquisition time of each volume.

Unlike traditional spatial encoding, the rapid acquisition of several low-resolution 3D volumes means that subject motion can be visualized throughout an imaging experiment. Where motion disturbs the steady state, the off-resonance profile deviates from the ideal peaked behavior, and super-resolution reconstruction will generate artefacts. This would be problematic for a straightforward reconstruction, but if the acquisition has redundancy in the collected data motion-corrupted volumes could either be corrected based on self-navigation or excluded entirely. This could provide a method of surpassing the pragmatic limits on spatial resolution imposed by the subtle motion of organs (in the presence of eg, vessel pulsation). We are currently exploring such models to increase the achievable spatial resolution in neuroimaging.

In conclusion, we have demonstrated that the off-resonance profile of the bSSFP sequence can be exploited to achieve high degrees of super-resolution enhancement with minimal RF power requirements. This opens up new opportunities for rapid MRI, particularly in high magnetic fields (≥3T).

## Supplementary Material

Supplementary material **FIGURE S1** Relative intensity of harmonics in a typical COMBINE acquisition with different tissue properties. Top) For a tissue with T_2_ = 80 ms, the harmonic intensities are asymmetric and negative components (ie, where *m* < 0) can be omitted from the reconstruction. Bottom) Where both T_1_ and T_2_ are long, the negative harmonics (*m* < 0) make a significant contribution to the acquired signal. This will cause aliasing during the reconstruction unless the number of acquired images, *N*, is increased
**FIGURE S2** Bloch simulations of the transient period of a bSSFP experiment in CSF (T_1_ = 2569 ms, T_2_ = 329 ms), using a single flip angle of either 1° or 30°. The plot shows the variation of signal magnitude at the center of the pass band in each case, ie, 0 Hz off-resonance for 1° and 1/2TR Hz off-resonance for 30°

## Data Availability

The phantom data that support the findings of this study are openly available at github.com/petelally/SuperOffRes along with a simple MATLAB (The MathWorks, Natick, MA) implementation of the multi-frequency super-resolution reconstruction algorithm.

## References

[1] Scheffler K, Lehnhardt S. Principles and applications of balanced SSFP techniques. Eur Radiol. 2003;13:2409–2418. 12928954 10.1007/s00330-003-1957-x

[2] Bangerter NK, Hargreaves BA, Vasanawala SS, Pauly JM, Gold GE, Nishimura DG. Analysis of multiple-acquisition SSFP. Magn Reson Med. 2004;51:1038–1047. 15122688 10.1002/mrm.20052

[3] Miller KL, Jezzard P. Modeling SSFP functional MRI contrast in the brain. Magn Reson Med. 2008;60:661–673. 18727099 10.1002/mrm.21690

[4] Shcherbakova Y, van den Berg CAT, Moonen CTW, Bartels LW. PLANET: An ellipse fitting approach for simultaneous T(1) and T(2) mapping using phase-cycled balanced steady-state free precession. Magn Reson Med. 2018;79:711–722. 28543430 10.1002/mrm.26717 PMC5811804

[5] Wood TC, Teixeira RPAG, Malik SJ. Magnetization transfer and frequency distribution effects in the SSFP ellipse. Magn Reson Med. 2020;84:857–865. 31872921 10.1002/mrm.28149 PMC7216875

[6] Henze Bancroft LC, Strigel RM, Hernando D, Utilization of a balanced steady state free precession signal model for improved fat/water decomposition. Magn Reson Med. 2016;75:1269–1277. 25946145 10.1002/mrm.25728 PMC5034725

[7] Lustig M, Santos J, Pauly J. A super-FOV method for rapid SSFP banding artifact reduction. Proc Intl Soc Mag Reson Med 13, 2005; Miami Beach, FL. Abstract 504.

[8] Gustafsson MG. Nonlinear structured-illumination microscopy: Wide-field fluorescence imaging with theoretically unlimited resolution. Proc Natl Acad Sci. 2005;102:13081–13086. 16141335 10.1073/pnas.0406877102 PMC1201569

[9] Gustafsson MG. Surpassing the lateral resolution limit by a factor of two using structured illumination microscopy. J Microsc. 2000;198:82–87. 10810003 10.1046/j.1365-2818.2000.00710.x

[10] Ropele S, Ebner F, Fazekas F, Reishofer G. Super-resolution MRI using microscopic spatial modulation of magnetization. Magn Reson Med. 2010;64:1671–1675. 20872866 10.1002/mrm.22616

[11] Hennel F, Tian R, Engel M, Pruessmann KP. In-plane “superresolution” MRI with phaseless sub-pixel encoding. Magn Reson Med. 2018;80:2384–2392. 29656440 10.1002/mrm.27209

[12] Hennel F, Pruessmann KP. MRI with phaseless encoding. Magn Reson Med. 2017;78:1029–1037. 27774644 10.1002/mrm.26497

[13] Zur Y, Stokar S, Bendel P. An analysis of fast imaging sequences with steady-state transverse magnetization refocusing. Magn Reson Med. 1988;6:175–193. 3367775 10.1002/mrm.1910060206

[14] Malik S, Sbrizzi A, Hoogduin H, Hajnal J. Equivalence of EPG and isochromat-based simulation of MR signals. In Proc Intl Soc Mag Reson Med 24. 2016; Singapore. Abstract 3196.

[15] Man LC, Pauly JM, Macovski A. Multifrequency interpolation for fast off-resonance correction. Magn Reson Med. 1997;37:785–792. 9126954 10.1002/mrm.1910370523

[16] Zhu J, Klarhöfer M, Santini F, Scheffler K, Bieri O. Relaxation measurements in brain tissue at field strengths between 0.35 T and 9.4 T. Proc Intl Soc Mag Reson Med 23, 2014.

[17] Stöcker T, Vahedipour K, Pflugfelder D, Shah NJ. High-performance computing MRI simulations. Magn Reson Med. 2010;64:186–193. 20577987 10.1002/mrm.22406

[18] Wansapura JP, Holland SK, Dunn RS, Ball WS Jr. NMR relaxation times in the human brain at 3.0 Tesla. J Magn Reson Imaging. 1999;9:531–538. 10232510 10.1002/(sici)1522-2586(199904)9:4<531::aid-jmri4>3.0.co;2-l

[19] Nishimura D, Vasanawala S. Analysis and reduction of the transient response in SSFP imaging. Proc Intl Soc Mag Reson Med 8, 2000; Denver, CO. Abstract 301.

[20] Setsompop K, Fan Q, Stockmann J, High-resolution in vivo diffusion imaging of the human brain with generalized slice dithered enhanced resolution: Simultaneous multislice (gSlider-SMS). Magn Reson Med. 2018;79:141–151. 28261904 10.1002/mrm.26653 PMC5585027

[21] Balezeau F, Eliat PA, Cayamo AB, Saint-Jalmes H. Mapping of low flip angles in magnetic resonance. Phys Med Biol. 2011;56:6635–6647. 21941028 10.1088/0031-9155/56/20/008 PMC3391187

