# Supplementary material for: Unbalanced SSFP for super-resolution in MRI
Source: Magn Reson Med. Author manuscript; Available in PMC 2022 Apr 1. (PMC8972796; doi:10.1002/mrm.28593)
Supplement: Supplementary material — FIGURE S1 Relative intensity of harmonics in a typical COMBINE acquisition with different tissue properties. Top) For a tissue with T2 = 80 ms, the harmonic intensities are asymmetric and negative components (ie, where m < 0) can be omitted from the reconstruction. Bottom) Where both T1 and T2 are long, the negative harmonics (m < 0) make a significant contribution to the acquired signal. This will cause aliasing during the reconstruction unless the number of acquired images, N, is increased FIGURE S2 Bloch simulations of the transient period of a bSSFP experiment in CSF (T1 = 2569 ms, T2 = 329 ms), using a single flip angle of either 1° or 30°. The plot shows the variation of signal magnitude at the center of the pass band in each case, ie, 0 Hz off-resonance for 1° and 1/2TR Hz off-resonance for 30° [file NIHMS1786935-supplement-Supplementary_material.doc]

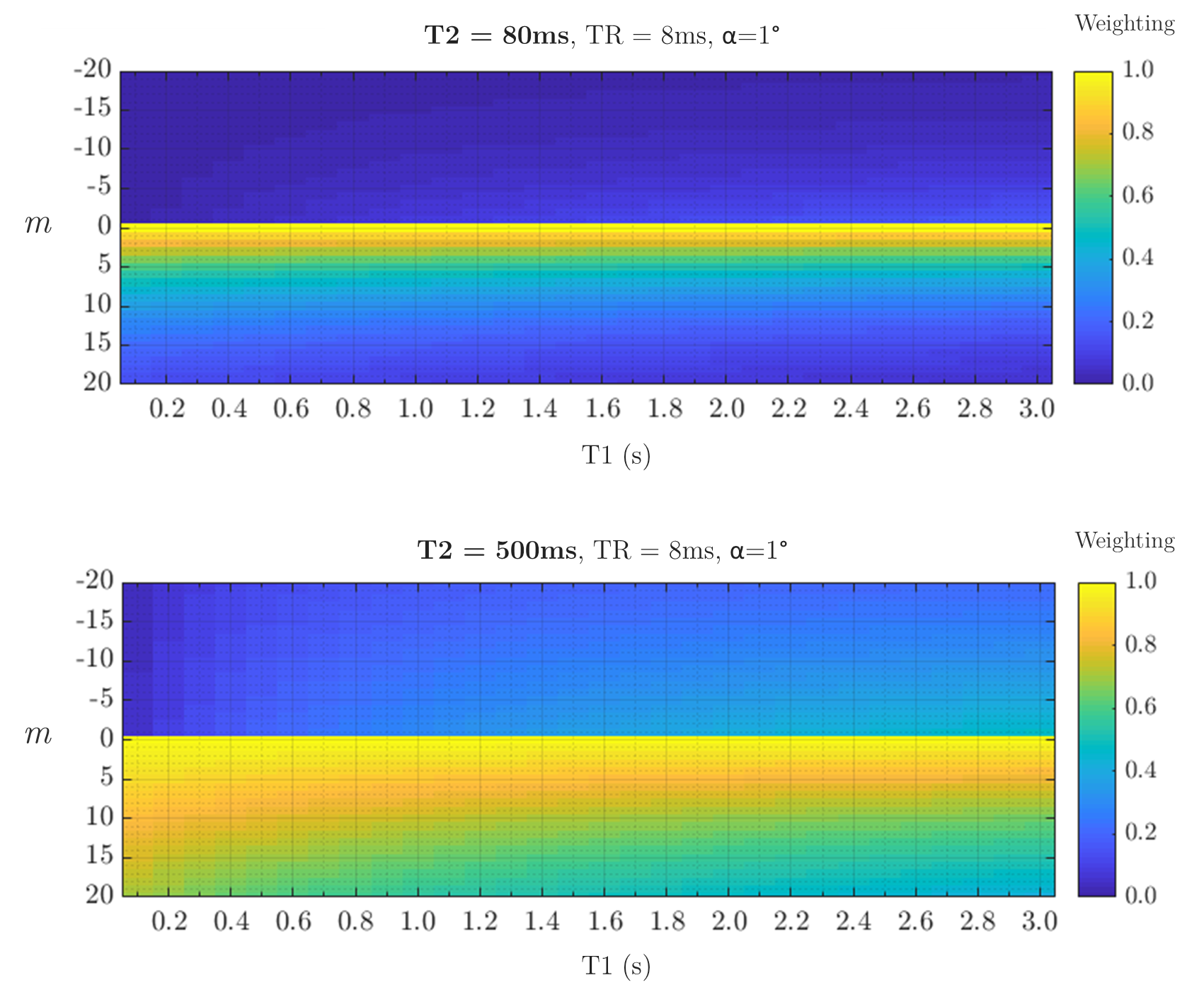


**Figure S1:** Relative intensity of harmonics in a typical COMBINE acquisition with different tissue properties. **Top)** For a tissue with T_2_=80ms, the harmonic intensities are asymmetric and negative components (i.e. where *m*<0) can be omitted from the reconstruction. **Bottom)** Where both T_1_ and T_2_ are long, the negative harmonics (*m*<0) make a significant contribution to the acquired signal. This will cause aliasing during the reconstruction unless the number of acquired images, *N*, is increased.


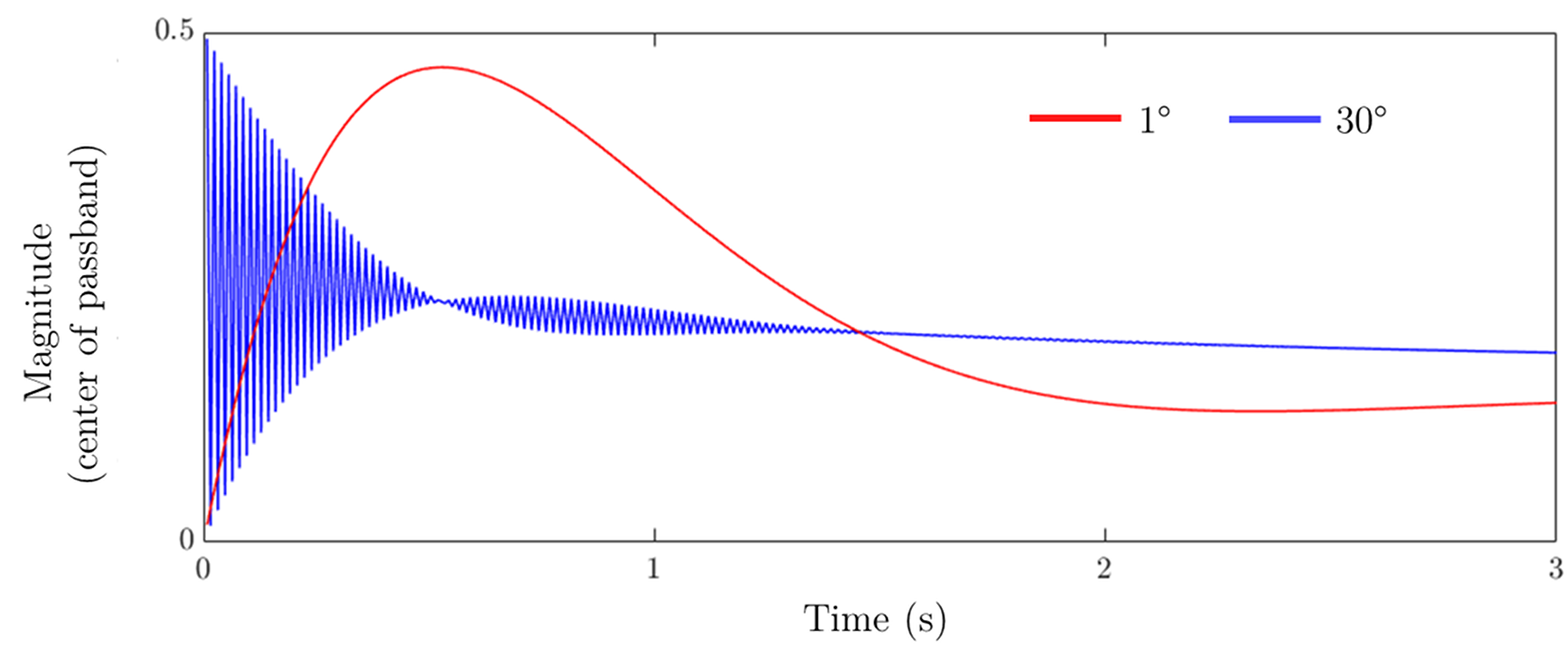


**Figure S2:** Bloch simulations of the transient period of a bSSFP experiment in CSF (T_1_=2569ms, T_2_=329ms), using a single flip angle of either 1° or 30°. The plot shows the variation of signal magnitude at the center of the pass band in each case, i.e. 0 Hz off-resonance for 1° and 1/2TR Hz off-resonance for 30°.
